# Law of Cholera

**Published:** 1854-11

**Authors:** 


Law of Cholera.—In our number for November, 1853, p. 189, we noticed a very important law in reference to the influence of the altitude of places on the mortality of this disease, demonstrated by Mr. Farr, in his reports on cholera in 1849. This law, which was established from data, afforded by observations in the London Basin, may be thus expressed: The increments of mortality in individual districts of a given spot, bear a certain constant relation to the order of the altitude of these districts. The experience of the past year, as shown by the report of the Register-General of England, confirms the truth of this law. Since the first calculations of Mr. Farr, Dr. Duncan, of Liverpool, has, at the request of the former gentleman, examined the influence of the elevation of soil on the increments of mortality from cholera in that city, and confirms Mr. Farr's law so far as to show that "when the districts of approximating elevations are grouped together, and the groups so formed contrasted, the results distinctly point to a relation between the elevation of the soil and the mortality from cholera." More general, though far less exact (mathematically speaking), observation fully confirms the truth of Mr. Farr's law. In whatever part of the globe the history of the cholera there ranging is investigated, it will be found as the rule that the disease affects most fatally the low lying seaports and deltas of rivers, sparing the high grounds, even round river sources. In India, we know it began in the delta of the Ganges, ravaged all low-lying places; while sepoy lines, placed from sixty to a hundred feet above the general level of the country, had scarcely any cases, excepting such as occurred in persons on guard at the different outposts. The higher classes of natives and Europeans generally inhabiting the better raised and more airy parts of the towns suffered proportionably less than the lower ranks, and indeed the general result was such as to lead Mr. Jameson, in his report of the Bengal cholera, in 1817–'19, to say that "there is abundant proof that in high, dry, and generally salubrious spots, it was both less frequent in its appearance, and less general and fatal in its attacks than in those that were low and manifestly unwholesome." In illustration of the law (established at any rate for the great "London Basin") that *the mortality from cholera is in the reverse ratio of the elevation*, we may remark, that if the districts of London are classified according to their elevation above the level of the Thames, in those whose elevation is not on an average twenty feet above its water line, it will be found that "on the bottom of the London Basin the mortality was at the average rate of 102 in 10,000; while in a district, 100 feet high, the mortality was 17 in 10,000; and in another (Hampstead), of an altitude of 350 feet, it was 8, or, deducting a stranger infected at Wandsworth, but who died there, 7 in 10,000." Now, if the mortality from cholera, according to the data here involved, and as occurring in relation to the elevation and mortality of other groups of districts included between those of less than 20 and above 350 feet of altitude be closely examined, the relation between elevation and death will be expressed as in the following table, on the one hand, as the facts actually occurred, and, on the other, as they should occur according to a series calculated from an equation worked out by Mr. Farr in his cholera report:

| Elevation of districts in feet above high-water. | Deaths in 10,000. Mortality observed. | Calculated series. |
|---|---|---|
| 20 feet | 102 | $\frac{102}{1}=102$ |
| 20— 40 | 65 | $\frac{102}{2}=51$ |
| 40— 60 | 34 | $\frac{102}{3}=34$ |
| 60— 80 | 27 | $\frac{102}{4}=26$ |
| 80—100 | 22 | $\frac{102}{5}=20$ |
| 100—120 | 17 | $\frac{102}{6}=17$ |
| 340—360 | 7 | $\frac{102}{18}=6$ |

It was found by Mr. Farr, on comparing the numbers of his series with the mean mortality observed in districts of eight different elevations, that the only considerable discrepancy was at the mean elevation (20—40) assumed to be thirty feet. The relation thus indicated being so important, it was thought proper by its discoverer to submit the principle to another test—viz: to compare the elevation and mortality from cholera of each *sub-district*, and this comparison, though it made the mortality on the lowest level less, and became deranged somewhat by the deaths in hospitals and workhouses, entirely confirmed the announced law. (*Report on Cholera*, p. lxvii.) Whilst the *inverse ratio* is, we think, generally amply sustained, it yet suffers, it must be confessed, considerable perturbation; so much so, in some cases, as to have led to but little appreciation of its value. "Instances of this kind," remarks Dr. Baly (College of Phys. Report, 18), "which are found to be very numerous, when the relation between the mortality from cholera and the elevation of the surface is examined in the cases of the sub-districts of the metropolis, go far to prove that, even in London, the connection between lowness of level and the prevalence of cholera, depends, at least, in great part on the character of the inhabitants and their dwellings in the lower districts, and on the foulness, as distinguished from mere dampness of the atmosphere in their localities." Dr. Duncan found also in Liverpool, that taking the districts singly, where the difference of elevation is only two or three feet, the law was not carried out, *being apparently overpowered by disturbing elements.* Notwithstanding all this, and much more which might be adduced if our space permitted, we accord in the opinion that, in considering the law thus announced, it is necessary to view it in connection with certain circumstances impeding its operation, and producing some deviation from strict mathematical accuracy, and that, such being yielded, as should be done in all complicated questions of hygiene, we shall find "that this inverse ratio is maintained as accurately as is requisite for the theory."—*Medical News and Library.*

